# Analysing the Quality of Life of Older Adults: Heterogeneity, COVID-19 Lockdown, and Residential Stability

**DOI:** 10.3390/ijerph191912116

**Published:** 2022-09-25

**Authors:** Ching-Yi Chen

**Affiliations:** Department of Living Science, National Open University, New Taipei City 247, Taiwan; chingyichen@mail.nou.edu.tw

**Keywords:** quality of life, heterogeneity, social isolation, COVID-19, residential stability

## Abstract

This study aims to, first, investigate the quality of life (QOL) of older adults in Taiwan, from the perspective of heterogeneity and, second, clarify the social isolation affecting their QOL during the pandemic. Additionally, it explores the residential stability of older adults. Our empirical model, a Generalized Ordered Probit Model, uses secondary data from the 2019 Taiwan Social Change Survey for people aged 65 and above, with a total sampling size of 417. The results show that the self-assessed physical health of older adults is a significantly heterogeneous variable affecting their QOL, and has a varying impact on the probability of them being satisfied with their QOL. Results suggest that better financial plans and staying healthy are the main determinants of their QOL. Social isolation during the COVID-19 pandemic significantly hampered their QOL, which was not compensated by the use of the internet. Furthermore, older adults’ residential stability significantly influences their QOL. Family members and relevant social work units should contribute to in-person home care for older adults to reduce their social isolation, with a focus on improving their living environments and to ensure that they age in a safe and healthy place.

## 1. Introduction

An assessment of the quality of life (QOL) of older adults helps to understand their awareness of well-being, capability for self-care, and daily life arrangements [1,2,3,4,5]. Older adults generally suffer from health issues and loss of functionality, which can cause inconvenience in their daily lives and deter social activity [4,6]. Some of them also face social isolation because of retirement [7]. Owing to this reduction in economic and social resources, they may perceive that their well-being is deteriorating [1].

The World Health Organization (WHO, 2022) [8] defines QOL as an ‘individual’s perception of their living situation, understood from a cultural and value system context, and in relation to the objectives, expectations, and standards of a given society’ [9]. However, there is no consensus in the literature on the definition of QOL [5,10,11,12]. Several terms are used in place of QOL, such as a satisfying life, subjective and psychological welfare, and personal development. Many researchers consider QOL as an indicator synonymous with a satisfying life and sense of well-being [13].

Looking at QOL from a broad perspective, Ven Leeuwen et al. (2019) use search terms such as ‘life satisfaction’, ‘successful aging’, ‘living well’, and ‘well-being’ to analyse the meaning of QOL among older adults [5]. However, they state that it is unclear how the domains identified in the study relate to other concepts of QOL, well-being, or successful ageing. Scharf et al. (2008), analysing the responses of participants belonging to focus groups in England’s socially deprived neighbourhoods [14], discovered that the participants faced difficulties understanding the phrase QOL and often mentioned health and finances. Rondón García and Ramírez Navarrro (2018) believe that QOL and life satisfaction are different but complementary concepts [13].

Given that there is no consensus on the definition or understanding of QOL among researchers and older adults, we find that older adults generally rely on their cognition and life contexts to explain QOL. Thus, it becomes difficult for researchers to know which life factors, situations, or dimensions equate to QOL for older adults, much less which policies or strategies can help improve their life.

QOL is an extremely abstract concept. Netuveli and Blane (2008) asked older adults about their QOL and found that they did not know precisely what it meant [15]. Compared to asking about QOL, other questions can be more specific, such as: How do you feel about your physical health? Are you satisfied with your life? Are your economic resources stable? Furthermore, studies show that factors such as physical health and functionality are more effective in predicting older adults’ QOL. However, the effect of other factors, such as exercise frequency, age, and marital status, is not consistent with QOL [16,17]. These findings highlight the heterogeneity in the evaluation of such subjective indicators as QOL.

Typically, an individual’s life experience and culture, along with their habits, social support, and relationships, significantly affect QOL [18]. As stated earlier, older adults answer questions on subjective factors based on their background, experiences and understanding of QOL, or depending on current situations (such as the pandemic-induced social lockdown). This dynamicity brings heterogeneity to their answers. Providing evidence for this observation, Scharf et al. (2008) show that poor older adults frequently equate the concept of QOL with their health and finances because these aspects govern their life experiences and contexts [14]. Thus, one cannot ignore the potential subjective heterogeneity in assessing the QOL of older adults.

Many studies discuss the QOL of older adults, but there has been no empirical research on its heterogeneity. However, researchers can present potent policies to improve the lives of older adults with an empirical model to estimate the heterogeneity in older adults’ QOL. Therefore, the purpose of this study is to, first, develop an empirical model to assess the QOL of older adults and explore its subjective heterogeneity so as to fill this gap in the existing research. Second, it investigates how the social isolation (from the COVID-19 pandemic) and residential stability have affected the QOL of older adults, which too has been under-researched. Thus, this study attempts to effectively extend the existing literature on older adults’ QOL, while providing solution strategies at the same time.

## 2. Literature Review

This section captures the critical determinants of the QOL of older adults based on the existing literature. In 2019, many countries prohibited public gatherings to control the spread of COVID-19. Similarly, until April 2022, Taiwan implemented a zero-COVID policy and relatively stricter control over social activities and gatherings. These actions resulted in extreme social isolation. Steptoe et al. (2013) show that social isolation reduces the size of social networks and creates a paucity of social contact [19]. It is also highly associated with health deterioration and mortality [20,21,22,23]. Pietrabissa and Simpson (2020) reveal that prolonged isolation adversely affects physical and emotional health, altering sleep and nutritional rhythms and decreasing opportunities for movement [24]. Consequently, the natural channels of human expression and pleasure become depressed, impacting mood and subjective well-being [25,26]. Netuveli and Blane (2008) state that high-quality social relations improve the QOL of older adults [27].

Based on these findings, social lockdown is a factor worth exploring. The prolonged social isolation during the pandemic affected psychological health, inducing stress, anxiety, and depression [28,29,30]. Such reduced in-person communication can negatively affect the QOL of older adults. Even though social media and online communication tools can compensate for in-person interactions by offering a new lifestyle [29], evidence of its effect on the QOL of older adults is scant. Furthermore, many older adults are either not good at, or do not like, using technological software. Thus, they suffer a more severe social isolation than others. This study, therefore, considers the number of people with whom an older adult interacts daily in-person and the frequency of using online social and communication software in assessing their QOL.

According to the WHO (1947), QOL is a vital measure of overall health. It captures the complete state of perceived physical and mental health and social well-being, not merely the absence of disease and infirmity [31]. Sleep quality, an indicator of health, plays a vital role in mental health, thus becoming a determinant of older adults’ QOL [32,33,34]. Several studies have shown that good sleep quality is associated with endocrine and cognitive function as well as psychological health [35,36]. It also reduces the chances of Alzheimer’s disease [36,37]. Complete rest helps people increase avidities of personal and social life [38]. Sleep problems in older adults are complex; they are caused by degeneration, mental state, and life habits. Some of the older adults have an inactive life schedule, with less physical activity or poor dietary habits, resulting in a lack of sleep. Thus, the sleep quality of older adults cannot be ignored while discussing QOL. Self-assessment based on physical health is vital to the perception of the QOL of older adults [5,14,15]. This study also considers older adults’ self-assessment of physical health as a determinant of their QOL in the empirical model.

Among all the determinants of older adults’ QOL, residential stability cannot be ignored; however, its effect has not been researched enough. Given a rapidly ageing population structure, a global phenomenon, older adults, along with their longevity, generally expect to spend their later years at home or in the same place [39,40]. Frequent moving in this age damages their psychological health and impairs their sense of coherence [41]. It has a detrimental impact on their daily life since their responses and memory gradually deteriorate, and it is hard for them to restore their social network. Ebrahimi et al. (2013) found five subthemes that affect the QOL of older adults [42]. Among them is remaining in familiar surroundings, which makes older adults feel secure and allows them to determine how to gauge or resist risks with ease and gain resources [43,44]. Furthermore, older adults ageing in the same place can show their competence and maintenance of a dynamic balance in their environment, which is conducive to maintaining a high QOL, sense of place, and psychological well-being [45]. A home represents much more than just a residence for the elderly because they are deeply connected to it and endeavour to stay in it as long as possible [42]. A home brings a familiar community-induced feeling of being in a pleasant place with familiar objects and shared norms and values [42]. Thus, this study considers the residential stability of older adults in the empirical model.

Finally, as mentioned before, participants have a poor understanding of what QOL means. They often refer to their health and financial conditions [14]. Additionally, since older adults are retired and their health may be poor, economic resource shortages are often a key determinant of their QOL. Netuveli et al. (2008) discuss longitudinal QOL at older ages [15]. Their findings indicated that the QOL can be reduced by poor financial status (β − 0.157). Thus, to capture the socio-economic status of older adults, this study focuses on their monthly income.

## 3. Methodology

### 3.1. Data Collection

For the analysis, this study uses secondary data obtained from the ‘2019 Taiwan Social Change Survey (Round 7, Year 5): Technology and Risk Society’, conducted by Academia Sinica. The survey was completed in February 2020 [46]. This was a national face-to-face survey that collected data through stratified and multi-stage probability sampling. With 1933 completed questionnaires, it had a completion rate of 48% [46]. In total, 417 data samples for were adults over 65 years old were collected for the analysis. Additionally, the survey period pertains to that of the COVID-19 pandemic; thus, there is a focus on social isolation among older adults.

### 3.2. Method

**Figure 1:**
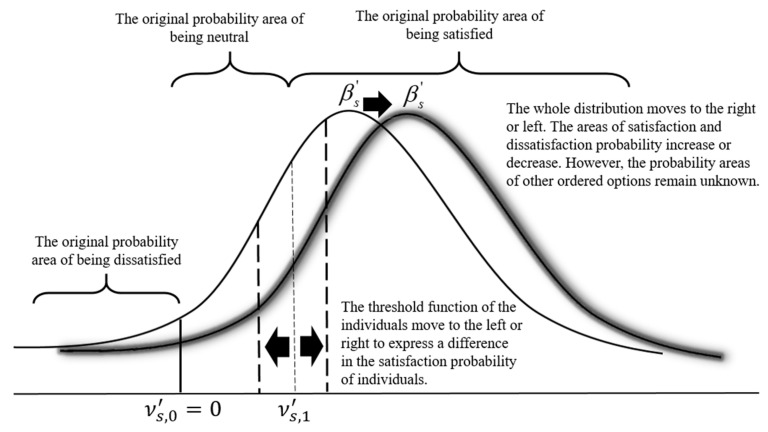
A schematic diagram of the conceptual GOP model of the parameter $\beta_{s}^{\prime }$ and the thresholds parameter $\nu_{s,1}^{\prime }$.

Since this study explores the QOL of older adults and focuses on its heterogeneity, a Generalized Ordered Probit (GOP) Model is used to account for this heterogeneity. This model is derived from the traditional Ordered Probit (OP) Model [47]. The dependent variable topic of this study is: Are you satisfied with your current quality of life? The outcomes include: Very Unsatisfied, Unsatisfied, Neutral, Satisfied, and Very Satisfied, conforming to the requirements of an OP model. The ordinal threshold values in the traditional OP model are fixed. Furthermore, the coefficient ($\beta_{s}^{\prime }$) represents the average selection of the samples regardless of the heterogeneity in their selection. It is calibrated and estimated using the potential function. Positive and negative values of $\beta_{s}^{\prime }$ indicate a right or a left shift in the whole normal distribution, respectively; that is, there is an increase or decrease in the probability areas of satisfaction and dissatisfaction. However, changes in the probability of other ordered options cannot be directly interpreted by the coefficient $\beta_{s}^{\prime }$ [48]. Figure 1 illustrates how a traditional OP model’s $\beta_{s}^{\prime }$ estimation would express older adults’ QOL. The ability of a traditional OP model to explain the QOL of older adults is quite limited.

Indeed, every individual sample should have different selections of the sequential probability areas. In other words, some people will exhibit a higher probability of satisfaction, while others will show a lower probability [49]. Since the QOL of older adults is a subjective measure, the cut-off point of the OP model’s threshold values involves the social and economic conditions of individuals and the impact of their life course. There will be individual demand differences, evaluations, and comparison benchmarks. Therefore, heterogeneity is considered. By assuming that the open thresholds are fixed, the GOP model accounts for the subjective difference in the satisfaction probability of individual respondents [50]. It also avoids the estimation error arising from neglecting the observable error term in the traditional OP model [47,50]. Figure 1 illustrates how a GOP model’s threshold function would estimate older adults’ QOL.

Equation (1) presents the mathematical formula of the traditional OP model. To obtain an equation for the GOP model, subscript *s* is added to the threshold values to form $\mu_{s}$, as shown in Equation (2). This equation indicates that the threshold values allow the changes arising from the differences in the selection of individuals [49,51].

(1) $$Qol_{s}^{*}=\beta_{s}^{\prime }X_{s}+\varepsilon_{s}$$

where $Qol_{s}^{*}$ is the satisfaction from QOL that cannot be observed by individual s;

$X_{s}$ is the explanatory variable vector for individual s;

$\beta_{s}^{\prime }$ is the parameter vector $X_{s}$ to be estimated;

$\varepsilon_{s}$ is a random error term that follows the standard normal distribution. Next,

(2) $$Qol_{s}=j,\;\mathrm{if}\;{\tilde{\mu }}_{s,\;j-1}<Y_{i}^{*}\leq {\tilde{\mu }}_{s,\;j},\;j=1,\;2,\ldots ,\;J,$$

where j is the observed ordinal variable, takes on values 1 through J.

The threshold parameter function range corresponding to each sample is $\left(-\infty <{\tilde{\mu }}_{s,\;0}<{\tilde{\mu }}_{s,\;1}<\ldots <{\tilde{\mu }}_{s,\;j}<\ldots <\infty \right)$. The threshold function of the respondents is expressed in Equation (3).

(3) $${\tilde{\mu }}_{s,\;j}={\tilde{\mu }}_{s,\;j-1}+e^{\eta_{s,\;j}+\nu_{s,\;j}^{\prime }k_{s,\;j}}$$

where $k_{s,\;j}$ is the vector of the variables;

${\tilde{\mu }}_{s,\;j}$ is the threshold value corresponding to the variable $k_{s,\;j}$;

$\nu_{s,\;j}^{\prime }$ is the parameter vector corresponding to $k_{s,\;j}$;

$\eta_{s,\;j}$ is the constant term of the threshold function.

The parameter threshold values in the GOP model are calibrated using the maximum likelihood method with the functional formula presented in Equation (4).

(4) $$LL=\sum {}_{s}Ln\{\Phi \left({\tilde{\mu }}_{s,\;j}-\beta_{s}^{\prime }X_{s}\right)-\Phi \left({\tilde{\mu }}_{s,\;j-1}-\beta_{s}^{\prime }X_{s}\right)\}$$

Parameters such as $\beta_{s}^{\prime },\;\eta_{s,\;j},\;\nu_{s,\;j}^{\prime }$ are also estimated using the maximum likelihood method. Moreover, the coefficient calibrated and estimated from the threshold variable ($\eta_{s,\;j},\;\nu_{s,\;j}^{\prime }$) is substituted into the overall model. The right or left movement of the threshold values’ cut-off point will increase or decrease the satisfaction probability areas, thus showing differences in individual behaviour and heterogeneous preferences [50].

### 3.3. Descriptive Statistics and Variables

Our sample comprises responses from 213 men (51.1%) and 204 women (48.9%) aged between 65 and 95 years. Based on their responses, 5 respondents (1.2%) are very unsatisfied with their QOL, 25 (6.0%) are unsatisfied, 74 (17.7%) are neutral on the subject, 241 (57.8%) are satisfied, and 72 (17.3%) are very satisfied. However, most respondents are satisfied with their QOL, and only a few are very unsatisfied and unsatisfied. After the pre-test, the threshold value between satisfied and very satisfied is found to be significant, indicating a significant difference in the individuals’ probability of selecting these outcomes. To focus on the threshold value of the significant part, this study reduces the variables to the third order, which is also more conducive to the convergence of the complex model. As a result, 30 respondents are unsatisfied (7.2%), 74 are neutral (17.7%), and 313 are satisfied (75.1%). This order conforms to the requirements of an OP model for our empirical analysis.

Table 1 presents the explanatory variables, and their symbols and response percentages. Based on the findings of previous studies, this study classifies the determinants of the QOL of older adults into their social situation, health and economic condition, and residential stability. In this empirical model, the older adult’s social situation is represented by the number of people with whom an older adult daily interacts in-person and the frequency of their use of social networking sites or communication software in the past year. Self-assessment of physical health, sleep quality, and physical and mental disorders of the older adult or their family members signify health conditions. Furthermore, residential stability refers to the duration of living in the same place and the monthly income denotes the economic situation.

A special focus was given to the existing literature’s claim that the Internet has become a new lifestyle after the pandemic. However, data on the frequency of social networking sites or communication software usage in the last year show that 72.9% of older adults stated they did not or would not use an Internet-based communication software. Therefore, while the new Internet-based lifestyle seems to have positively impact the young and middle-aged, it may not have worked for the older generation. The experience and skill gap faced by the older generation should not be ignored.

## 4. Empirical Results

Table 2 shows the empirical results of executing the GOP model. This study segregated the vital variables affecting QOL of older adults into two models to avoid mutual obstruction resulting out of the presence of multiple variables in a pool model. Model 1 focuses on the impact of social situation, self-assessment of physical health, sleep quality, and residential stability of the older adults on their QOL. This study aims to understand the effect of a social lockdown on older adults. Model 2 captures the influence of the physical and mental disorders, and economic conditions of respondents or their family members on their QOL. Literature review suggests the latter to be a crucial affecting variable. It must be considered that the respondents had retired and perhaps lacked adequate funds. Additionally, some older adults, or their family members, might fall ill or suffer from a debilitating illness or disability. This might pose a challenge and compel them to make a life adjustment, which in turn might affect their QOL.

### 4.1. Social Situation

Model 1 shows that the coefficient is −0.424 when older adults interact in person with less than four persons daily. This negative value will shift the whole normal distribution to the left, indicating an increase in the probability area of very unsatisfied with the QOL. It also implies that too few daily in-person interactions make older adults highly dissatisfied with their QOL. Furthermore, the coefficient of the frequency of using social websites or communication software is not significant, indicating that online interpersonal interactions do not affect older adults’ QOL.

### 4.2. Health Condition

The self-assessed health of older adults is a significantly heterogeneous variable in Models 1 and 2. The coefficients ($\nu_{s,1}^{\prime }$) are −0.998 and −1.012, respectively, indicating that when the threshold value moves to the left, the probability area of being satisfied with the QOL increases; that is, the higher the self-assessed health of older adults, the more satisfied they feel with their QOL. This finding shows a heterogeneity among individual samples. Self-assessed poor health is also a statistically significant heterogeneous variable. The coefficients are 0.402 and 0.300 for Models 1 and 2, respectively, indicating that when the threshold value moves to the right, the probability area of being neutral-satisfied increases and that of being satisfied reduces. It demonstrates that those who assessed their health as poor are more likely to feel neutral-satisfied with their QOL.

Regarding sleep quality, the coefficient of poor sleep quality in Models 1 and 2 are −0.365 and −0.408, respectively, and is statistically significant. The coefficients indicate that when the whole normal distribution shifts to the left, the probability area of being unsatisfied with the QOL increases. This finding implies that improving sleep quality of older adults increases their satisfaction with their QOL. In Model 2, the coefficient of the number of daily in-person contacts is not significant after considering the physical and mental disabilities of respondents or the family members living with them. However, the coefficient of the number of mobility-impaired family members living with the respondent is −0.476 and is statistically significant. This finding indicates that when the whole normal distribution shifts to the left, the probability area of being satisfied with the QOL decreases. It demonstrates that older adults will be significantly unsatisfied with their QOL when a family member, such as a spouse, has mobility issues. This result should be a wake-up call since there are many households in Taiwan where people both young and older live with and care for the elderly members. Therefore, it is very important for the family to maintain good health and avoid accidental injuries, which will increase older adults’ satisfaction with their QOL.

### 4.3. Residential Stability

In Models 1 and 2, the coefficients are −1.237 and −1.371, respectively, when the older adults stayed at the current place for less than three years. This finding indicates that when the whole normal distribution significantly shifts to the left, the probability area of being unsatisfied with the QOL increases. It demonstrates the importance of living in a familiar place for older adults in their later years of life and highlights that ageing at home or the same residence/locality helps maintain their QOL.

### 4.4. Economic Condition

The coefficients of the monthly income between 20,000 and 40,000 TWD and exceeding 40,000 TWD in Models 2 are 0.647 and 1.137, respectively, and are statistically significant. This finding indicates that when the whole normal distribution shifts to the right, the probability area of being satisfied with the QOL increases. Thus, the QOL satisfaction threshold income for older adults is 20,000 TWD (approximately USD 667). Older adults with a monthly income more than 20,000 TWD are more satisfied with their QOL.

## 5. Discussion and Implications

This section discusses the critical determinants of the QOL of older adults based on the empirical results, including the significant heterogeneity variables, social isolation, and residential stability. With regard to the heterogeneity status of the QOL of older adults, healthy older adults have a significantly larger probability area of being satisfied with their QOL than those who do not identify as physically healthy. Healthy older adults’ threshold value shifted to the left, increasing the probability area of satisfaction to 0.8944.

Conversely, the threshold value shifted to the right for those who assessed their health as poor. The probability area of satisfaction reduced from 0.8591 to 0.8428, and the probability area of being neutral increased to 0.1317. The heterogeneity of the two variables, self-assessed good health and self-assessed poor health, suggests that the physical health of older adults creates a significant difference in the probability area of satisfaction with the QOL. The GOP model revealed a heterogeneity among older adults, which addresses the gap in the literature on the heterogeneity of their QOL. This finding highlights the importance of maintaining good physical health for older adults as it boosts their independence, self-esteem, and QOL, thus benefiting their family and society.

Another vital finding is that older adults’ satisfaction with their QOL reduces if they live with a mobility-impaired family member, such as a spouse or parent. Together, the two findings imply that older adults’ satisfaction with their QOL is significantly diminished when their physical health is poor or they must take care of a mobility-impaired family member. Therefore, suitable policies must be in place that ensure that older adults have adequate knowledge about healthcare, such as knowledge on healthy diets for preventing chronic diseases. In addition, older adults must be educated and encouraged to remodel their house as needed to avoid physical injuries to household/family members. Moreover, the government, including urban planning departments, should focus on providing safe and friendly public spaces to older adults. It should construct community parks and pedestrian walkways for exercise in outdoors or public spaces. It should also emphasise building infrastructure that helps to minimise falls, accidents, or vehicle collisions. Furthermore, when it comes to caring for older adults, the focus should shift from providing bed rest to providing a healthy, active, and stimulating environment that gives older adults an opportunity to stay healthy and prevent mental and physical disabilities. This step is particularly important since many countries are on way to being super-ageing societies. In a developed country, a healthy indoor and a friendly outdoor living space for the twilight years are essential. These improvements are beneficial for the national welfare policy and reduce the burden on government and family care.

The empirical results also show that older adults’ dissatisfaction with their QOL increases when they interact in person with fewer than four people daily. This finding supports the results of Steptoe et al. (2013) [19]. Furthermore, the lockdown significantly and negatively impacted the daily life of even healthy and active older adults, and affected their QOL. This finding aligns with that of Colucci et al. (2022) and Prati (2020), who showed that quarantine during the pandemic negatively affected perceived health and well-being [52,53]. Additionally, this study verifies that the frequency of using social websites or communication software does not affect older adults’ QOL significantly.

Before the pandemic broke out, many countries and communities implemented meal-sharing policies for older adults or provided public spaces so they could play, chat, and indulge in other social activities. However, such activities were banned during the pandemic, greatly impacting older adults’ daily life, activities, and sense of coherence. While online communication has become popular as a new mode of interaction, results indicate it cannot replace or compensate for the lack of older adults’ physical social activities. Thus, family members and relevant social services should actively provide in-person home care to older adults to reduce the adverse effect of social isolation.

This study also confirms that a less-than-three-year stay at their current home exerts a significantly negative impact on older adults, decreasing their likelihood of being satisfied with their QOL. Older adults desire a familiar living environment. Ageing at home or at the same location is also a salient elderly care policy in developed countries. This finding corresponds to those of Burns et al. (2012) [54] and Kaplan et al. (2015) [55], who showed that a familiar living environment enables older adults to obtain a consistent sense of life and maintain their habits [54,55]. Moreover, older adults have a high degree of residential inertia, and relocation disturbs their social and living habits. Older adults and family members should focus on improving their indoor living environment looking to the future, such as by reducing the number of stairs and increasing handbars. The government may provide more residence- or community-improvement subsidies, such as subsidies for improving sidewalks or adding elevators in apartment buildings, to improve older adults’ living situations. Such measures will aid older adults in a super-ageing society and reduce the negative impact on their QOL. Improving their living environment will be conducive to maintaining their physical and mental health, thus reducing the burden on the government to care for older adults.

## 6. Conclusions

This study verifies that measuring the QOL of older adults is synonymous with evaluating their self-assessed physical health. It also reveals that their physical health creates heterogeneity in their QOL. This finding implies that, when measuring their satisfaction with their QOL, it is preferable to provide clear definitions to reduce subjectivity.

The study also explores how the prohibition of in-person social activities and the frequency of online communication impacted the QOL of older adults during the pandemic. Essentially, restrictions on in-person social interactions hamper their health, and online social activities cannot compensate for the lack of in-person social interactions. Furthermore, maintaining residential stability enables older adults to live in a familiar, healthy, and safe place and continue their daily habits, which is conducive to having a high level of QOL. Deteriorating physical and psychological functions gradually limit their range of activities, and it is not easy for them to adapt to a new living environment.

Data and research on how social isolation affects the life or psychology of older adults are still insufficient. Future studies should extend the investigation on the impact of social isolation on older adults’ QOL during the pandemic and use a clear definition of QOL to conduct surveys. This will facilitate the development of more appropriate measures and policies to reduce any adverse impacts on their QOL.

## Figures and Tables

**Table 1:** Explanatory variables, symbols, and response percentages.

Variable	Symbol	%
**Number of persons with whom daily in-person interaction takes place**		
Less than 4 persons	Pfour	43.50
5 to 9 persons	Pfive	23.50
More than 9 persons	Pother	33.00
**Frequency of using social websites or communication software during the most recent year**		
Several times a day	Esocialt	14.60
Almost every day	Esociald	12.50
Other	Eother	72.90
**Self-assessment of physical health**		
Very good	Hwell	43.50
Poor	Hbad	23.50
Other	Hother	33.00
**Sleep quality in the past year compared to that in the previous year**		
Slightly bad (including much worse)	Sleepb	34.50
Good	Sleepg	10.80
Other	slother	54.70
**Physical and mental disorders of respondents and their family members**		
A mobility-impaired family member lives with the respondent	Fdisable	17.30
No mobility-impaired family member lives with the respondent	Fdisother	82.70
The respondent is holding the disability card	Indisablec	8.60
The respondent is not holding the disability card	Indisother	91.40
The respondent lives with family members who hold the disability card	Fdisablec	10.30
The respondent lives with family members who do not hold the disability card	Fdisother	89.70
**Residential stability**		
Since birth to the present	Liveo	12.90
Less than three years	Liveless	1.70
Other	Lother	85.40
**Total monthly income**		
Less than 10,000 TWD (around USD 333) (including those who mentioned zero and who refused to answer the question)	Income1	49.60
10,000 to less than 20,000 TWD (around USD 333)	Income2	21.30
20,000 to less than 40,000 TWD (around USD 1333)	Income3	16.80
More than 40,000 TWD	Income4	12.30

**Table 2:** The results of executing the GOP model.

Variable	Model 1		Model 2	
	$\hat{\beta }$ Value (T Value)	$\nu_{s}^{\prime }$$\;\mathrm{Value}\;\mathrm{of}\;{\tilde{\mu }}_{s,\;1}$ (T Value)	$\hat{\beta }$ Value (T Value)	$\nu_{s}^{\prime }$$\;\mathrm{Value}\;\mathrm{of}\;{\tilde{\mu }}_{s,\;1}$ (T Value)
Const.	1.774 *** (11.388)	−0.063 (−0.471)	1.866 *** (9.315)	0.052 (0.690)
**The number of persons with whom daily in-person interaction takes place**				
Pfour	−0.424 * (−2.165)	N/A	−0.266 (−1.265)	N/A
Pfive	−0.156 (−0.970)	N/A	−0.070 (−0.407)	N/A
Pother	Reference Group			
**Frequency of using social websites or communication software during the most recent year**				
Esocialt	−0.015 (−0.070)	N/A	−0.324 (−1.371)	N/A
Esociald	0.499 (1.567)	N/A	0.413 (1.209)	N/A
Eother	Reference Group			
**Self-assessment of physical health**				
Hwell	N/A	−0.998 *** (−3.601)	N/A	−1.012 *** (−3.712)
Hbad	N/A	0.402 ** (2.715)	N/A	0.300 * (2.048)
Hother	Reference Group			
**Sleep quality in the past year compared to that in the previous year**				
Sleepb	−0.365 * (−2.503)	N/A	−0.408 ** (−2.696)	N/A
Sleepg	0.072 (0.285)	N/A	0.146 (0.561)	N/A
Slother	Reference Group			
**Physical and mental disorders of respondents and their family members**				
Fdisable	N/A		−0.476 * (−2.546)	N/A
Fdisother	Reference Group			
Indisablec	N/A		0.385 (1.445)	N/A
Indisother	Reference Group			
Fdisablec	N/A		−0.346 (−1.607)	N/A
Disother	Reference Group			
**Residential stability**				
Liveo	−0.270 (−1.388)	N/A	−0.276 (−1.378)	N/A
Liveless	−1.237 ** (−2.588)	N/A	−1.371 ** (−2.858)	N/A
Lother	Reference Group			
**Total monthly income**				
Income1	N/A		−0.099 (−0.587)	N/A
Income3	N/A		0.647 * (2.474)	N/A
Income4	N/A		1.137 ** (2.995)	N/A
Income2	Reference Group			
Sample size	417			
Statistical				
LL(β)	−256.7828		−239.3280	
BIC	552.8676		525.8184	
CAIC	604.0977		587.2946	

*, **, and *** indicate that the two-tailed test results are significant below the 5%, 1%, and 0.1% levels, respectively. ‘N/A’ indicates that the variable is not included in the model.

## Data Availability

The data presented in this study are openly available in [46].
